# Free-floating thrombus in the aortic arch

**DOI:** 10.1590/0100-3984.2016.0083

**Published:** 2017

**Authors:** Marcelo Coelho Avelino, Carla Lorena Vasques Mendes de Miranda, Camila Soares Moreira de Sousa, Breno Braga Bastos, Rafael Soares Moreira de Sousa

**Affiliations:** 1 Hospital de Urgência de Teresina Prof. Zenon Rocha, Teresina, PI, Brazil; 2 Med Imagem, Teresina, PI, Brazil; 3 UDI 24 horas, Teresina, PI, Brazil; 4 Hospital Antônio Prudente, Fortaleza, CE, Brazil

**Figure 1:**
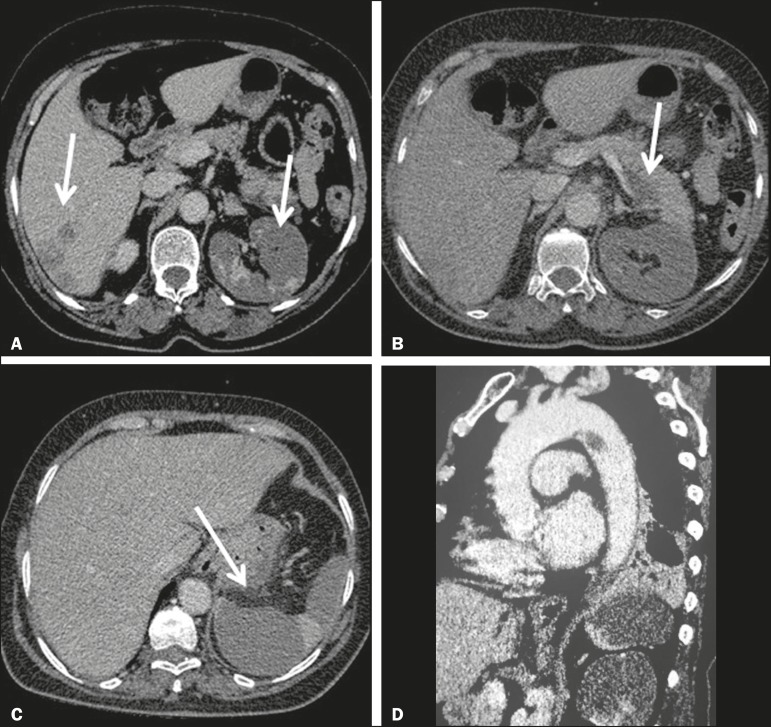
Contrast-enhanced axial CT of the abdomen, axial sections, showing areas of low uptake, characteristic of infarcts, located in the right lobe of the liver (**A**) and in the body of the pancreas (**B**), partially involving the left kidney (**A**) and spleen (**C**). D: CT angiography of the thoracic aorta showing a pedunculated thrombus adhered to the distal portion of the aortic arch, on the wall opposite the origin of the subclavian artery.

*Dear Editor*,

A 32-year-old female patient was admitted to the emergency room with abdominal pain, nausea, and vomiting. During the diagnostic investigation, she reported no history of comorbidities. A contrast-enhanced computed tomography (CT) scan of the abdomen demonstrated areas of low uptake, characteristic of infarcts, located in the right lobe of the liver and in the body of the pancreas, partially involving the left kidney and spleen, accompanied by luminal filling defects in the celiac trunk and its branches, as well as in the left renal artery and superior mesenteric branches, consistent with thrombi (Figures 1A, 1B and 1C). Laboratory tests revealed hypercoagulability due to protein C deficiency. The investigation was complemented with CT angiography of the thoracic aorta, which revealed low-attenuation material within the lumen, with a pedicle adhered to the distal portion of the aortic arch, on the wall opposite the origin of the subclavian artery, resulting in a filling defect, suggestive of pedunculated thrombus (Figure 1D). We considered the possibility of a free-floating thrombus in the aortic arch, complicated by systemic embolization. Surgical removal of the thrombus, with repair of the base of the lesion, was indicated. Subsequently, histopathology confirmed the initial hypothesis of an organized thrombus, with no evidence of malignancy.

Free-floating thrombus in the aorta is defined as a nonadherent portion of a thrombus, floating within the aortic lumen. It is a rare condition, only approximately 100 cases having been described in the literature. In general, it is uncommon to find an aortic thrombus in the absence of an aneurysm or atherosclerosis^(1)^. In such cases, the thrombus is often associated with hypercoagulability, trauma, malignant neoplasm, previous surgery, or turbulent blood flow.

The clinical presentations of free-floating thrombus range from asymptomatic disease to symptoms related to cerebral, peripheral, or organ embolization^(1-6)^. However, the prevalence of embolization is higher in cases of floating thrombi than in those of adherent thrombi (75% vs. 12%). Although most cases of aortic thrombus are diagnosed after embolic events, some are diagnosed on the basis of incidental findings on routine examinations^(1,5)^. As reported in the literature, the diagnosis has typically been confirmed through transesophageal echocardiography or CT angiography, the latter being the modality that is currently preferred^(1)^. Among thrombi in the thoracic aorta, the most common locations are the aortic isthmus and the distal portion of the aortic arch on the side opposite the origin of the subclavian artery^(4)^.

Because of the high risk of massive systemic embolization, it is considered necessary to treat a free-floating thrombus in the aortic arch. However, the ideal treatment remains undefined. Although the use of a thrombolytic is considered one of the options, it carries the risk of selective lysis of the pedicle of the lesion, which would have catastrophic results. In selected patients, surgical treatment is thought to be the most acceptable option^(2,4-6)^.

The purpose of this report was to describe a rare case of floating thrombus in the aortic arch with systemic embolization. In the case reported here, the thrombus was treated successfully through surgery.
